# Processed EEG Artifacts and Pitfalls: A Narrative Review and a Troubleshooting Mnemonic

**DOI:** 10.7759/cureus.103656

**Published:** 2026-02-15

**Authors:** Alexandre Pinheiro, Vasyl Katerenchuk, Alexandre M Calçada, Rita Regufe, Ana C Batista, Irene Ferreira

**Affiliations:** 1 Anesthesiology, Unidade Local de Saúde da Arrábida, Setúbal, PRT

**Keywords:** depth of anesthesia monitoring, eeg artifacts, eeg interference factors, electroencephalography, electroencephalography (eeg), processed eeg

## Abstract

Processed electroencephalography (EEG) is widely used for depth of anesthesia monitoring. However, multiple interference sources can distort raw EEG data, leading to misinterpretation of quantitative EEG (qEEG) indices, potentially resulting in insufficient or excessive depth of anesthesia or burst suppression. This review summarizes qEEG interference and artifact sources, as well as some paradoxical effects and pitfalls, reported in the literature. These factors can be categorized into pharmacological, electromagnetic and mechanical, pathophysiological, and model-related sources. When unexpected qEEG values lack an immediately apparent explanation, a structured approach can be valuable, particularly in complex circumstances. We provide a relatable mnemonic “ANESTHESIA” for troubleshooting potential interference sources. If artifact removal is impractical, this should be acknowledged when adjusting the anesthetic management plan. A systematic approach to identify qEEG artifacts and acknowledge potential pitfalls may improve the reliability of depth of anesthesia monitoring, ultimately enhancing patient safety.

## Introduction and background

Depth of anesthesia (DoA) monitoring through electroencephalography (EEG) has transformed anesthetic management in recent decades. It corroborates adequate intraoperative hypnosis, allows anesthetic dose titration, and prevents excessive DoA [1,2]. The EEG records mainly cortical electrical activity through electrodes placed typically on the frontal scalp. The EEG signal comprises a complex waveform that can be automatically analyzed and decomposed into its constituent wave frequencies and amplitudes, which can be associated with different states of consciousness [2-5]. Many proprietary algorithms have been developed, which analyze several aspects of processed frontal EEG, providing a quantitative EEG (qEEG) index value representing DoA [1-5].

The appealing simplicity of qEEG has encouraged its use in routine practice, yet sole reliance on its numerical value has several pitfalls, since multiple interference sources are known to skew raw EEG data, potentially leading to wrongful interpretation of DoA [1,4]. This can lead to anesthetic dose titration based on an incorrect assessment of DoA, risking deepening of anesthesia in patients who are already adequately anesthetized, increasing the risk of burst suppression and postoperative delirium. Conversely, misinterpretation of the current DoA state can also lead to a reduction of anesthetic dosage in adequately anesthetized patients, thereby increasing the risk of traumatic intraoperative awareness.

Acknowledging these interference factors is essential for an accurate assessment of qEEG reliability, contributing to a safer anesthetic management [6,7].

This narrative review summarizes qEEG interference and artifact sources, as well as some paradoxical effects and pitfalls, reported in the literature. Our objective is to provide anesthesiologists with a practical, mnemonic-based framework, "ANESTHESIA," to identify and troubleshoot artifacts in processed EEG monitoring.

## Review

Methods

This article was designed as a narrative review. We searched the PubMed® (MEDLINE) database to identify papers written in English, reporting or documenting qEEG interference factors, published from inception until August 2025. The searched combinations of terms included the following: “depth anesthesia artifacts”, “depth anesthesia monitoring”, “EEG depth of anesthesia limitations”, “bispectral index interference”, “BIS artifacts”, “EEG artifacts anesthesiology”, “electromyography bispectral index”, and “clinical EEG monitoring anesthesiology”.

All retrieved articles were initially screened by title. The final selection of studies for inclusion was performed based on an analysis of the abstracts and a subjective assessment by the authors regarding their pertinence and correspondence to the review’s objectives. While the search prioritized literature reporting or documenting qEEG interference factors, references considered relevant for clarifying foundational concepts of EEG monitoring were also included. As a limitation to our search strategy, some articles were excluded due to the unavailability of the full text.

Electroencephalography and depth of anesthesia monitoring interpretation

The clinical significance of each frontal EEG frequency band in a density spectral array (DSA) has been discussed in the literature by numerous authors [3,4,6]. In summary, the awake state is characterized by the detection of high-frequency and low-amplitude waves like beta (13-25 Hz) and gamma (> 26 Hz) waves [3,6]. Sedation induced by GABAergic agents is characterized by an increased alpha (9-12 Hz) and beta wave power [3,6]. Under GABAergic general anesthesia (GA), a predominance in high-amplitude and lower frequency waves like slow (<1 Hz), delta (1-4 Hz), and alpha waves is seen [3,6]. In deeply anesthetized patients, slow-delta waves predominate, and burst suppression may occur [3,4,6,8].

The various EEG monitors available rely on proprietary algorithms to analyze multiple descriptive factors from EEG data, in order to calculate the qEEG, which ranges from 100 (awake state) to 0 (isoelectric EEG) [2,3,6,9].

As an example, even though the Bispectral Index™ monitoring system (BIS™) proprietary algorithm is able to filter out multiple artifacts, several factors still frequently alter the EEG signal, resulting in a skewed qEEG value [5,10,11]. Furthermore, a specific qEEG value will not necessarily correspond to the same DoA, depending on the agent being administered [3,4,6]. This highlights the importance of evaluating all aspects of EEG monitoring, not just the index value. For example, anesthetic agents produce distinct EEG and DSA signature patterns that should be incorporated into our global assessment [3,4,6]. Even brief tutorials enable anesthesiologists to achieve a reasonable degree of competence in EEG interpretation [6,12].

In this review, we categorized known interference factors into the following categories: pharmacological, electromagnetic and mechanical, pathophysiological, and model-related factors.

Pharmacological factors

Intravenous Anesthetics and Adjuvants

Ketamine blocks inputs to inhibitory interneurons, allowing disinhibition of downstream excitatory neurons in the cortex and other subcortical structures [3]. This causes increased beta-gamma and decreased delta wave power, while also increasing desynchronization in EEG patterns [3,4]. Consequently, this results in significant qEEG increase, despite a deeper DoA, in patients under a GABAergic GA (e.g., sevoflurane or desflurane) [6,13,14].

Dexmedetomidine leads to an increase of inhibitory inputs to the arousal centers and decreases excitatory inputs to cortical and some subcortical structures [3,6]. It produces an EEG pattern that resembles stage II or III of non-rapid eye movement sleep patterns, characterized by spindle-like alpha waves and slow-delta waves [3,6]. These EEG characteristics can result in lower qEEG, consistent with a deepening of DoA, in patients under GA [15]. However, a lower qEEG is also possible even under moderate sedation [3,16]. Clonidine produces similar effects on qEEG [17].

An opioid-free strategy combining low-dose esketamine and dexmedetomidine, under sevoflurane GA, produces higher qEEG and spectral edge frequency 95% (SEF95) values, in comparison to opioid-based GABAergic maintenance strategies [18].

Inhaled Anesthetics

Nitrous oxide (N2O) mostly produces no significant effect on qEEG values in patients under GABAergic GA [1,4,5,19]. Nevertheless, a pattern characterized by increased beta-gamma wave power and a relative decrease in slow-delta wave power is typical after suspension of GABAergic agents with transition to high-flow N2O in the emergence phase [3]. Conversely, sudden suspension of N2O may paradoxically decrease the qEEG by augmenting low-frequency waves (withdrawal-suppression phenomenon) [1,4]. A clinically non-significant decrease of qEEG is also possible when N2O is added to sevoflurane anesthesia, although not through a hypnotic effect detection. The second-gas effect and improved analgesia could be a potential explanation for this finding [19].

An increase in isoflurane concentration may increase alpha-beta wave power and paradoxically elevate the qEEG [1,4]. Conversely, isoflurane and sevoflurane may induce a paradoxical low-voltage EEG phenomenon during rapid washout, leading to erroneously lower qEEG and burst suppression [1,4,6].

Halothane is typically associated with higher BIS index values at equivalent doses to other modern anesthetic vapors like sevoflurane; however, the BIS™ algorithm has not been validated for this agent [1,4].

Neuromuscular Blocking Drugs and Reversal Agents

Electromyography (EMG) activity produces artifacts within the gamma frequency range, potentially elevating the qEEG [1,4-6,19-22]. Neuromuscular blocking drug (NMBD) administration frequently leads to a qEEG reduction by diminishing EMG interference, even in awake patients [1,4-6,22]. This finding urges cautious interpretation, as NMBD suppresses movement, potentially compromising awareness recognition [6,19]. Succinylcholine is the only NMBD potentially associated with a qEEG increase, immediately after fasciculation [19,23]. This effect might be independent of EMG activity [19].

NMBD reversal agents, such as sugammadex and neostigmine, often lead to higher qEEG, mostly due to increased EMG activity-related artifacts [5,24,25]. Furthermore, an arousal effect after administration of sugammadex, in patients under propofol GA, has been studied, not only based on qEEG values but also clinical evaluation [24]. This effect can be relevant when sugammadex is administered at shallower DoA states [24]. Potential mechanisms like increased sensory afferent inputs to the arousal centers (“afferentiation”) and low-affinity propofol-binding properties have been previously suggested, but require further investigation [24].

Opioids

Analgesic doses exert minimal effects on qEEG, as their primary action involves non-cortical structures, even though they potentiate the anesthetic’s hypnotic effect [1,4,6,26,27]. If higher opioid and lower anesthetic doses are titrated, a higher qEEG is expected [1,4,6,26,27]. However, opioids may produce a significant qEEG decrease if high doses, superior to analgesic requirements, are administered [4,19]. It is uncertain if this occurs independent of hemodynamic variations, thus requiring further study [19].

Neuraxial Blockade and Local Anesthetics

Neuraxial blockade can produce a significant qEEG decrease [19,28]. Several mechanisms have been proposed, including systemic absorption, neuraxial rostral spread of local anesthetics, and loss of afferent inputs to the arousal centers [19,28]. Neuraxial blockade also decreases hypnotic agent requirements under GA [29].

Other Drugs

Numerous drugs have been previously reported to potentially influence the EEG trace and qEEG values, with variable clinical significance. Adrenergic drugs like epinephrine, ephedrine, noradrenaline, and isoproterenol, but not phenylephrine, have been reported to potentially elevate the qEEG [1,6]. The underlying mechanism is not yet well understood. A temporary but sudden and relevant reduction of qEEG can be seen after administration of successive doses of methylene blue [1]. Magnesium sulphate infusions have been demonstrated to lower qEEG values in obstetric patients under volatile GA, with sevoflurane or desflurane, in a double-blind randomized controlled trial [30]. Neuroleptics may induce either slow or fast EEG activity, resulting in variable effects on qEEG [6]. Aminophylline was reported to increase the qEEG, although the mechanism remains unclear [19]. Alcohol and drug use can also produce variable effects on the EEG. Chronic abuse of alcohol typically increases beta wave power [6]. The use of cocaine and cannabis has been associated with increased alpha wave power [6].

Electromagnetic and mechanical factors

Electrical Impedance

To maintain high signal quality, it is important to keep a low impedance and a high signal-to-noise ratio. This can be compromised by temperature, humidity, poor sensor quality, and low conductivity of head tissue [1,4,6,9,19,31,32]. Most manufacturers recommend abrasive skin preparation techniques prior to electrode application, which is often neglected in clinical practice [19].

Electromagnetic Interference

Electromagnetic interference is a major source of artifacts that may decrease signal quality. Many devices produce an electrical current simulating high-frequency EEG waves, like pacemakers. Other implantable cardiac devices, high-frequency diathermy, and EMG tracheal tubes have also been found to potentially elevate the qEEG [1,4,6,9,10,19,20,32,33]. Facial neuromuscular blockade monitoring inadvertently produces such artifacts, even when in standby mode, due to impedance testing [5,6,10,19,20,32]. Some devices create an electromagnetic field, which disrupts the EEG signal with high-frequency waves, especially when operated around the patient’s head (e.g., ENT navigation system), falsely increasing qEEG [1,4,6,7,31].

Cardiac Electrical Conduction System

The ECG signal is another physiological source of EEG trace artifacts, which the BIS™ proprietary algorithm mostly filters out [5,10,11]. However, in rare circumstances, the ECG signal may predominate over the EEG signal. This may occur in deeply anesthetized patients or in the presence of a low-voltage EEG phenomenon, which can be genetically determined or associated with previous severe brain injury or brain death, potentially elevating the qEEG [1,4,7,11].

Mechanical Vibrations

Forced-air warming blankets placed near the patient’s head can simulate high-frequency waves [1,4,7,9,31-33]. Other commonly used devices like surgical endoscopic shavers, Doppler ultrasound, and pulsatile cardiopulmonary bypass pumps can also produce such artifacts that cause a qEEG increase [1,4,7,19,31,33]. Despite this, it is concerning that a high signal quality index (SQI) value is often maintained, hindering accurate interpretation [1,4,9,20,21,31-33]. Arterial pulsation can also cause detectable mechanical vibrations, especially when a dynamic superficial temporal artery pulse is present [10,11].

Pathophysiological factors

Age

Processed EEG index algorithms were originally developed based on adults with an EEG considered normal, therefore requiring caution when applied to age extremes [1,3,8,34,35].

For pediatric patients, especially under one year of age, DoA monitors can display lower pre-awakening qEEG values and lower correlation of qEEG values with end-tidal concentration values of volatile anesthetics [36]. However, the correct interpretation of DSA patterns is useful to overcome this limitation [37].

Elderly patients exhibit a natural shift into a slightly faster and more irregular EEG wave composition [8,35,38]. There are age-related changes in alpha wave power, displaying a more significant decrease in power in comparison to slow wave power, therefore decreasing the alpha-to-slow ratio, leading to elevated qEEG, even at anesthetic concentrations of GABAergic agents [35]. They also exhibit lower power across all frequency bands and an increased likelihood of burst suppression, while generally maintaining the same DSA band patterns associated with GABAergic agents [8,35].

Nociceptive Stimuli

Nociceptive stimuli have variable effects and may produce a qEEG increase through cortical arousal [4,6,19,39]. Inadequate intraoperative analgesia may increase anesthetic dose requirements during strong nociceptive stimuli, which may also reflect in higher qEEG [4,19]. In this case, the proper titration of analgesia will lessen this qEEG increase [4]. However, a paradoxical reduction of qEEG, driven by an unexpected increase in frontal delta waves in response to nociception, has also been reported [1,6,39]. Garcia et al. have reviewed the effects of nociceptive stimuli on the EEG during GA. In their review, they described eloquently three types of nociception-induced changes in the EEG: “beta arousal,” characterized by an increase in beta wave power; “delta arousal,” characterized by an increase in slow-delta wave power; “alpha dropout,” characterized by a sudden reduction in alpha wave power [39].

Low-Voltage EEG Trace

A genetically determined low-voltage EEG baseline, found in 5%-10% of the population, may lead to inaccurate algorithmic evaluation, due to a flat EEG waveform [1,4,7,34]. Hence, the qEEG and EEG baseline trace should be checked prior to induction of GA [1,6,19]. A rare paradoxical qEEG reduction due to low EEG amplitude has also been reported during the elimination phase of remifentanil, isoflurane, and sevoflurane [1,4,6].

Cerebral Ischemic Events

A qEEG decrease is expected during global cerebral ischemia secondary to cardiopulmonary arrest, but it may also be observed due to more localized cerebral ischemic events [1,4,6,7,11,19]. In the latter case, however, it may be significantly more challenging to accurately identify the cause intraoperatively.

Temperature and Metabolic Activity

Hypothermia reduces anesthetic requirements and may significantly decrease the qEEG [1,4,6]. Several mechanisms based on pharmacokinetic changes at low temperatures have been described, such as increased inhaled anesthetic solubility in the lipid membrane or through a decrease in propofol biotransformation [4,6]. Hypoglycemia can also result in decreased cortical metabolic activity and is associated with an increase in slow-delta wave power, therefore decreasing the qEEG [1,4,6,19].

Seizure Activity and Post-ictal Phase

The high-frequency EEG bursts seen during seizure activity increase the qEEG [6]. The post-ictal phase after electroconvulsive therapy is characterized by a typical slow-delta wave pattern, resembling profound DoA. This pattern may be present even when the patient is recovering consciousness [1,4,6]. This phenomenon is not dependent on the anesthetic agent used [4].

Hemodynamics and Patient Positioning

Cerebral cortical activity is inextricably linked to cerebral perfusion [1,19]. Significant hemodynamic deviations from baseline, particularly episodes of severe hypotension accompanied by a sudden decrease in qEEG, may reflect compromised cerebral perfusion [1,19].

Maintaining a prolonged Trendelenburg position can lead to the development of forehead edema, which can potentially reduce the EEG signal amplitude [40]. Furthermore, sudden shifts in position (Trendelenburg or head-up position) may induce hemodynamic and cerebral perfusion variations associated with qEEG changes [19].

Brain Space-Occupying Lesions

Brain tumor patients seem to display a higher qEEG on induction with propofol, in comparison with spine surgery patients [19]. Additionally, qEEG values may also be discrepant between sides in patients with a unilateral lesion [19]. A frontal pseudomeningocele may produce an artifact signal similar to an ictal EEG discharge, due to mechanical vibrations produced by cerebrospinal fluid pulsation [10].

Neurological Disorders

Although current EEG processing algorithms have been developed based on a healthy population, neurological disorders may significantly affect the EEG signal. Alzheimer’s disease, vascular dementia, schizophrenia, and organic delusional states have all been found to increase slow wave power and decrease high-frequency waves while awake [1,4,6]. Therefore, it is not surprising that qEEG algorithms inaccurately display lower qEEG values in this group. Cerebral palsy children generally also display significantly lower qEEG in comparison to healthy children [1,4,6].

Model-related performance

Significant discrepancies in performance between different monitoring systems have been documented. This has been reported not only across different models produced by a specific manufacturer, but also between different monitoring systems created by distinct manufacturers [1,4,19,21]. Discrepant qEEG values for the same DoA may result from differences in algorithmic processing between models, or differences in the artifact detection or filtering capabilities of each model.

Anesthesiologists who apply different monitoring systems in different settings should consider that performance between models may vary [1,4,19,21]. This implies an inherent learning curve whenever a new model is used, requiring additional caution in qEEG interpretation.

Implications for clinical practice

**Figure 1:**
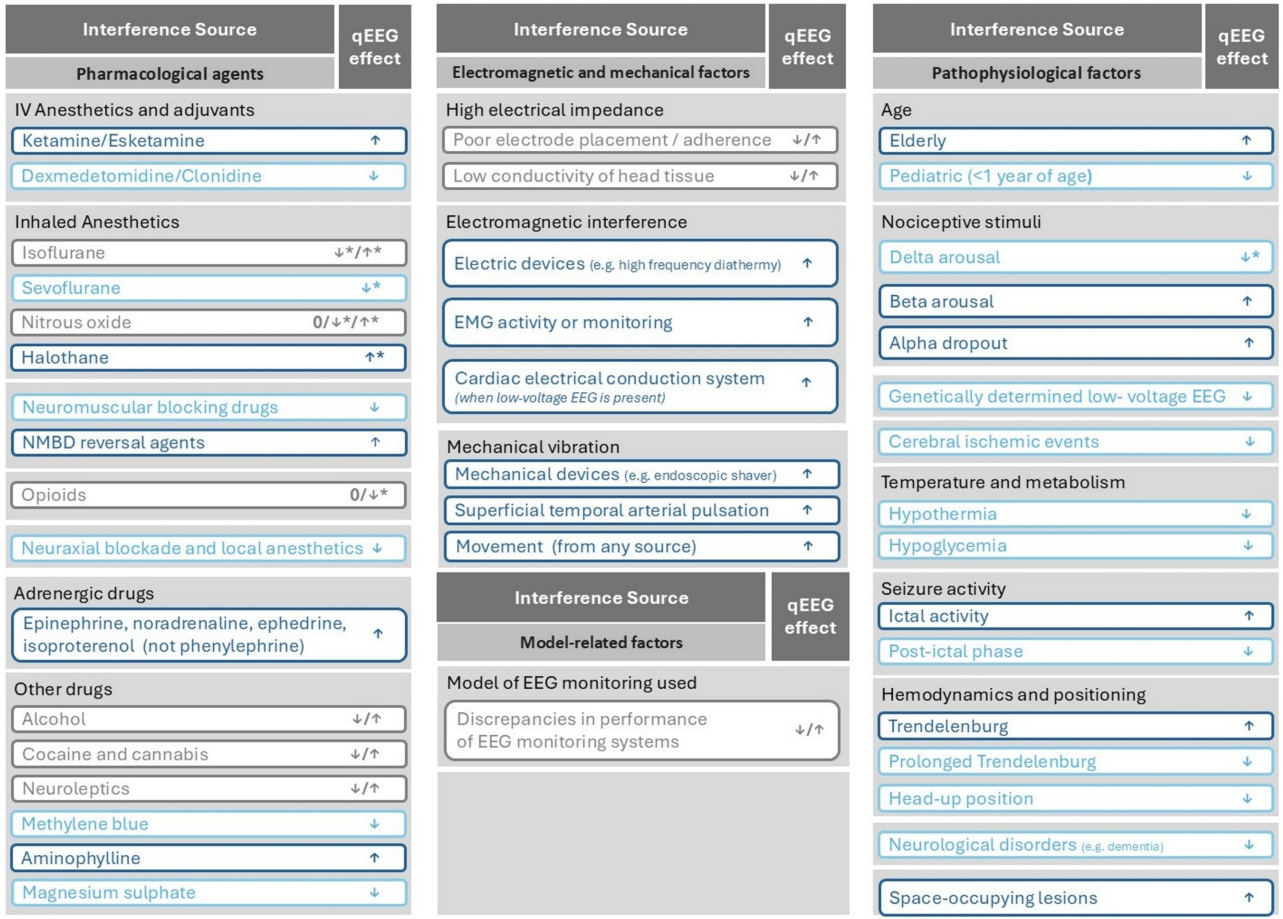
Summary of qEEG interference factors and their potential effects on qEEG index values. EEG: electroencephalography; IV: intravenous; NMBD: neuromuscular blocking drugs; qEEG: quantitative EEG; EMG: electromyography. * May produce a paradoxical effect under specific circumstances. References: [1,3-11,13-40]. Image credit: Alexandre Pinheiro and Vasyl Katerenchuk.

The interference factors and pitfalls discussed in this review (summarized in Figure 1) underscore the limitations of processed qEEG for DoA assessment and should be considered when troubleshooting potential qEEG discrepancies (unexpected or unjustified very low or high qEEG for a given clinical context). A more robust evaluation requires integration of multiple parameters, including qEEG value trend, DSA, EEG trace, SEF95, SQI, EMG measurements, patient comorbidities, as well as recent intraoperative events, whether they may be anesthesia or surgery related [1,6]. This multi-parameter approach provides a more comprehensive and nuanced understanding of the patient's neurophysiological state, enhancing the reliability of DoA assessment [1,6,7,10].

It is crucial to identify the source of interference to avoid causing harm to the patient due to artifacts or a malfunction of monitoring equipment, whether through excessive or insufficient DoA [1,6,7,10].

A troubleshooting mnemonic for qEEG discrepancies

Anesthesiologists should regularly monitor for potential qEEG discrepancies [6]. If a discrepancy is detected and an obvious justification is not immediately apparent, a systematized troubleshooting process may be helpful, especially under stressful or complex circumstances. Bennett et al. described a set of useful questions that help guide this process, mainly centered around the location of the source of interference [6]. We provide a simple, easy-to-relate mnemonic, “ANESTHESIA,” as a tool to overview all aspects of potential EEG interferences and pitfalls, which can easily be applied in clinical practice. However, this tool does not replace clinical judgment, which remains fundamental to the identification and management of any problems related to this issue [6]. Not every unexpected qEEG value or DSA pattern will correspond to an artifact or interference.

Since context is crucial for adequate assessment, some basic information should be quickly recollected regarding the patient’s medical comorbidities and age, as well as current information displayed on the DoA monitor, such as qEEG value, SQI, DSA pattern, EEG and SEF95 trace tendencies, EMG signal, and burst-suppression indicators. Under GA, if a sudden, unexpected qEEG increase is detected, anesthesiologists should search for faster wave power increase or slower wave power decrease. Conversely, if a qEEG decrease is detected, anesthesiologists should search for slower wave power increase, burst suppression, or a sudden decrease in faster wave power [4,6,37]. This information will be relevant for an adequate troubleshooting process of qEEG discrepancies.

Table 1 presents a set of troubleshooting questions, based on the mnemonic “ANESTHESIA,” designed to cover most clinically relevant sources of EEG interference. The goal is not to rigidly address every question in every scenario but to leverage this tool to prioritize issues most pertinent to a specific clinical scenario.

**Table 1:** The "ANESTHESIA" mnemonic checklist for identifying potential sources of EEG interference and pitfalls. DSA: density spectral array; EEG: electroencephalography; EMG: electromyography; IV: intravenous; MAC: minimum alveolar concentration; qEEG: quantitative EEG index; TCI: target-controlled infusion. References: [1,3-11,13-40]. Table credit: Alexandre Pinheiro and Vasyl Katerenchuk.

Mnemonic	Troubleshooting question
A (Anesthetic agents and other drugs)	General questions
	1. Are the EEG trace and DSA signature congruent with anesthetic agents currently being administered?
	2. What was the baseline qEEG value and DSA pattern before induction of general anesthesia?
	3. Was there any recent drug administered with a potential effect on EEG trace and qEEG?
	Under volatile general anesthesia
	1. What is the current end-tidal concentration and age-adjusted MAC value of a volatile anesthetic?
	2. If a paradoxical sudden qEEG decrease is detected on the emergence phase, was the volatile agent (or remifentanil) recently abruptly suspended?
	Under total intravenous anesthesia (TIVA)
	1. Is the syringe pump functioning properly?
	2. What is the TCI-target value and its corresponding rate of infusion?
	3. Is there drug extravasation, backward flow, or IV line occlusion?
N (Neuromuscular blockade and reversal agents; neurological disorders)	1. What is the current depth of neuromuscular blockade and EMG activity index value?
	2. Was a neuromuscular blockade reversal agent recently administered?
	3. Are there any known neurological conditions?
E (Equipment)	1. Is the signal quality index (SQI) low?
	2. Are EEG electrodes and cables adequately placed and connected according to the manufacturer’s guidelines?
	3. Can excessive sweat/humidity be contributing to the skin's low conductivity?
S (Stimulus)	1. Is there currently any significant nociceptive stimulus?
	2. Do other clinical signs or a nociception monitor index suggest inadequate analgesia?
	3. Are there EEG patterns associated with nociception?
T (Temperature and metabolism; tumors)	1. Is the patient severely hypothermic?
	2. Are there significant metabolic disturbances?
	3. Is there a documented frontal brain space-occupying lesion?
H (Hemodynamics and positioning)	1. Is there a significant hemodynamic deviation from baseline, potentially compromising cerebral perfusion?
	2. What vasoactive drugs are currently being administered?
	3. Can the current surgical positioning potentially affect cerebral perfusion or venous drainage, or promote forehead edema?
E (Electromagnetic and mechanical interferences)	1. Are there mechanical vibrations or movements originating from the surgical equipment or table?
	2. Are there electromagnetic interferences originating from the surgical equipment or an implantable cardiac device?
	3. Is there any device placed near the patient’s head that could produce electromagnetic or mechanical interference?
S (Seizure)	1. Is there a documented history of seizure episodes or disorders?
	2. Were anti-convulsive or pro-convulsive drugs administered or suspended in the perioperative period?
	3. Do the EEG trace and DSA display high-frequency EEG bursts that suggest an active seizure episode?
I (Ischemia and injury)	1. Are there cardiovascular high-risk factors for perioperative major adverse cardiovascular events?
	2. Does cerebral oximetry monitoring suggest a compromise of cerebral perfusion (if available)?
A (Age)	Children
	1. Is the patient an infant (under 1 year of age)?
	2. Is the DSA pattern consistent with adequate depth of anesthesia?
	Elderly
	1. Is there a baseline relative alpha power reduction under general anesthesia, or was it sudden?
	2. Is the current DSA pattern consistent with adequate depth of anesthesia when age-adjusted?

A - Anesthetic Agents and Other Drugs

An inadequate delivery of anesthetic agents should be excluded as a priority prior to investigating other potential sources of interference. This aims to recognize significant inadvertent under- or overdosing of anesthetics by clinical standards, minimizing risks and complications associated with both scenarios.

Congruency between the EEG trace, the DSA signature, and the specific agents being administered should be verified [6]. Distinct DSA signature modifications to GABAergic anesthesia baseline patterns can be induced after administration of specific agents (e.g., ketamine or N2O) [1,3,4,6,13-15]. Anesthesiologists should consider the potential influence of other medications (e.g., high-dose opioids, adrenergic drugs, and magnesium sulphate infusions), which may also influence the EEG trace [1,4,6,19,30]. This is particularly relevant for explaining sudden qEEG discrepancies that correlate with recent drug administration.

A quick review of the baseline qEEG index values and DSA signature obtained, prior to induction of GA and at the onset of the maintenance phase, should be performed, providing the necessary reference frame for accurate assessment [1,6,19].

The integrity of the anesthetic delivery system must be verified to systematically rule out technical failure. In the context of volatile anesthesia, this entails checking the end-tidal concentration and age-adjusted minimum alveolar concentration (MAC) value to ensure proper dosage of anesthetic.

During total intravenous anesthesia (TIVA), an inspection of the infusion system aims to detect syringe pump malfunctions, incorrect target-controlled infusion (TCI) parameter settings, or issues with intravenous (IV) access, such as line occlusion, kinking, disconnection, drug extravasation, or backward flow [41]. Pressure alarms should prompt an immediate inspection regarding these issues. Compliance with safety recommendations of TIVA administration as proposed by the Association of Anaesthetists and the Society for Intravenous Anaesthesia in their joint guidelines should reduce the frequency of complications related to infusion delivery [41]. For instance, the use of anti-reflux valves to prevent backward flow, if more than one infusion is given through a single IV cannula, as well as ensuring IV lines and cannulas remain visible, whenever practical, are particularly relevant measures regarding the aforementioned IV line issues [41]. Suspicion of IV cannula dislodgment or malfunction should be addressed immediately.

During the emergence phase, a sudden paradoxical decline in qEEG should be interpreted with caution; this may reflect a paradoxical withdrawal-suppression phenomenon after abrupt cessation of remifentanil or volatile agents, rather than a return to a deep hypnotic state [1,4,6].

Finally, unspecific clinical signs of insufficient DoA, such as spontaneous movement or an autonomic sympathetic response (most notably, but not exclusively, manifested by hypertension and/or tachycardia), require cautious interpretation and must be integrated into a multi-parameter assessment of DoA [6,39].

N - Neuromuscular Blockade and Reversal Agents; Neurological Disorders

Another priority is to assess the neuromuscular blockade (NMB) status, as this is crucial information to consider. Proper NMB monitoring equipment should be available and used if an NMBD is administered. If either spontaneous movement, a high EMG index, or a sudden increase of gamma waves is detected, EMG activity could be a potential justification for high qEEG. Administration of an adequate dose of an NMBD would help exclude EMG as a potential interference source. However, this should only be considered if clinically justified and desirable in a given clinical context, to avoid unnecessarily exposing the patient to the potential risks of NMBD. In patients under deep NMB, a sudden increase of beta-gamma waves could represent potential awareness, but other artifacts should also be considered and excluded [6,39].

The administration of NMB reversal agents, such as sugammadex, is frequently associated with qEEG increase and thus warrants cautious interpretation [5,24,25]. Usually, NMB reversal agents are administered at the end of surgery, which is usually accompanied by suspension of anesthetic agents, and so a qEEG increase would be expected anyway. However, recognizing this artifact becomes more relevant under specific circumstances, where reversal agents may be considered during the maintenance phase for neuromonitoring purposes (e.g., EMG, tracheal tube, or motor evoked potentials monitoring).

Additionally, pre-existing neurological disorders must be acknowledged, as they may significantly modulate EEG activity. Neurodegenerative conditions, such as Alzheimer’s disease, and cerebral palsy, a neurodevelopmental disorder, are typically associated with lower qEEG values; therefore, establishing a baseline is crucial both before induction and at the onset of the maintenance phase [1,4,6]. Given the sustained influence these disorders exert on the EEG trace, they are unlikely to cause transient or sudden qEEG discrepancies.

E - Equipment

The reliability of qEEG is intrinsically linked to signal integrity, which must be confirmed via the SQI and impedance testing [1,6,9,19]. Most commercial monitors possess an automatic impedance check function [6,19]. A suboptimal SQI should prompt a re-evaluation of the patient-sensor interface to rule out inadequate skin preparation, electrode displacement, or the accumulation of sweat and humidity [6]. Additionally, the integrity of all cable connections should be verified. It is worth noting that performance may vary between different commercial monitoring systems [1,4,19,21].

S - Stimulus

Intense nociceptive triggers can cause breakthrough nociception and lead to cortical arousal if analgesia is insufficient [4,6,19,39]. Therefore, the intensity of current surgical stimulus must be assessed while screening for specific EEG nociception-associated features, such as “beta arousal,” “alpha dropout,” or a paradoxical “delta arousal” [39]. If any of these features coincide with intense nociceptive stimuli, the observed qEEG discrepancy is likely attributable to nociception.

Nevertheless, since EEG monitoring is still considered a poor nociception monitoring method, commercial nociception monitors may be considered, if available, providing useful information to better correlate recent DSA changes with a response to nociception [6,39].

As previously discussed, interpreting unspecific clinical signs, such as hypertension or tachycardia, as a sympathetic autonomic response to nociception or insufficient DoA, requires careful consideration and must be integrated into a multi-parameter assessment of DoA and analgesia [6,39].

T - Temperature and Metabolism; Tumors

Temperature monitoring should be performed following both the American Society of Anesthesiologists (ASA) standards for basic anesthetic monitoring and local protocols. In most cases that typically include monitoring and active heating measures, severe hypothermia is not to be expected. However, in specific circumstances where significant hypothermia is induced, such as during interventions performed under cardiopulmonary bypass or deep hypothermic cardiocirculatory arrest, a significant reduction in qEEG may be detected [1,4,6]. Notwithstanding, if a false increase in qEEG is observed under deep hypothermic cardiocirculatory arrest, this may be attributable to pulsatile vibrations originating from the cardiopulmonary bypass pump, rather than a phenomenon related to temperature’s effect on the EEG trace [19].

Point-of-care testing, such as arterial blood gas analysis, can be performed to exclude major acid-base and electrolyte disturbances, as well as hypoglycemia, as potential sources of unexpected qEEG variations [1,4,6,19].

As mentioned previously, intracranial tumors or space-occupying lesions can produce EEG signal artifacts [19]. Since this is a rare form of artifact source, a high degree of suspicion is needed in patients with known brain tumors that are reported to produce artifacts like frontal pseudomeningoceles [10]. Nevertheless, these should not be responsible for sudden qEEG discrepancies, but rather a more persistent modification of the EEG baseline, which may also be apparent through a significant asymmetry between sides, when bilateral EEG monitoring is used [19].

H - Hemodynamics and Positioning

Cortical activity is intrinsically dependent on cerebral perfusion [1,19]. Major hemodynamic deviations from baseline, particularly severe hypotension accompanied by a sudden decrease in qEEG, are suggestive of cerebral hypoperfusion [1,19]. The appropriate therapeutic intervention must be tailored to the underlying cause of hemodynamic compromise.

Additionally, extreme surgical positioning (either Trendelenburg or anti-Trendelenburg positions) may impact cerebral perfusion pressure and indirectly qEEG values through different mechanisms [19,40]. Since these mechanisms are contingent upon positioning, a return to a neutral position should restore baseline values. While theoretically relevant to acknowledge this as a potential interference factor, reverting patient positioning solely to confirm its interference on EEG would be disruptive to surgical workflow and is typically unnecessary in most clinical scenarios, except when the patient does not tolerate such positioning because of hemodynamic compromise.

In cases of prolonged steep Trendelenburg, the resolution of physiological changes, such as venous congestion and localized forehead edema, after restoring a neutral position may occur more gradually, although Hoshi reported a rapid increase of qEEG after restoring neutral position and suspension of anesthetic delivery in one case report [40]. In this context, if a low qEEG and delayed emergence occur, we suggest excluding typical etiologies, such as metabolic disturbances, residual anesthetic or analgesic effect, residual NMB, or hypothermia, and following local protocols for neurological examination and neuroimaging studies, as these may be necessary to investigate potential cerebral ischemia or other structural lesions.

E - Electromagnetic and Mechanical Interferences

The reliability of qEEG is intrinsically linked to the EEG signal’s integrity, which can be compromised by numerous electromagnetic sources in the operating room [1,4,6,19,20,32,33]. External interferences originating from electrical devices, especially near the patient’s head, should always be considered. Diathermy is an easily identifiable and frequent source of electromagnetic interference; however, it also represents a typical source of nociceptive stimulus. If the considerations previously discussed regarding anesthetic dosage, analgesia, and NMB assessment have been addressed, then an increase in qEEG index values right after a prolonged application of diathermy can safely be discarded as an electromagnetic artifact. In this case, a significant distortion of the EEG trace can typically be seen during diathermy [1,6,19]. Nevertheless, if diathermy is responsible for qEEG discrepancies, then a return to qEEG baseline values is expected when its use is suspended. If this does not occur, other mechanisms of EEG distortion must then be considered. Some equipment frequently used under specific circumstances, such as facial nerve stimulators, EMG endotracheal tubes, or ENT navigation systems, can induce rhythmic artifacts that distort the EEG trace [1,4,6,7,31,32]. Similarly, mechanical interferences are a frequent source of error. Vibrations transmitted through the surgical table or generated by forced-air warming blankets placed near the patient’s head can mimic high-frequency oscillations of brain activity [1,4,9,11]. A common characteristic of these artifact sources is their contingency upon device operation, whereby interference can be effectively mitigated by deactivating or removing the source equipment. However, as these artifacts are typically transient, actions for their removal may be impractical or disruptive to surgical workflow and frequently unnecessary. Nevertheless, acknowledging the potential for inaccurate qEEG values remains essential for adequate assessment of DoA and anesthetic titration.

S - Seizure

Sudden high-amplitude spikes or rhythmic high-frequency bursts of EEG activity across the spectrum may signal an ongoing seizure, especially in patients with a documented clinical history of epilepsy or those with epileptogenic brain lesions [6,19]. In this case, local protocols regarding diagnostic and therapeutic approaches to perioperative seizure episodes should be followed. Clinicians should verify whether anti- or pro-convulsant medications were recently suspended or administered.

If low qEEG indices are observed during the post-ictal phase (e.g., following electroconvulsive therapy), cautious interpretation is necessary since the expected qEEG increase may lag behind recovery of consciousness in some cases [1,4,6].

I - Ischemia and Injury

Cerebral ischemia represents a critical differential diagnosis for a sudden, abrupt decline of qEEG values. This consideration is particularly relevant in patients with significant cardiovascular comorbidities or those undergoing procedures associated with a high risk of major cardiovascular events. In the absence of a recent anesthetic bolus, “delta arousal” or acute hemodynamic instability, a precipitous reduction in index values could represent localized acute cerebral hypoperfusion [4]. Integration with other forms of monitoring, such as cerebral oximetry based on near-infrared spectroscopy (NIRS), if available, could help to corroborate this hypothesis. As discussed earlier, if a cerebral ischemic event is suspected, then a neurological examination and neuroimaging studies should be performed, in accordance with local protocols, to confirm this diagnosis.

A - Age

Age is a significant influencing factor of the EEG trace, as mentioned previously. For pediatric patients, especially those under one year of age, an integrated assessment with other parameters (e.g., DSA patterns and end-tidal value under volatile anesthesia) can be used to help overcome the algorithmic limitations of qEEG monitors [37]. De Heer and Weber described a decision algorithm for propofol and sevoflurane GA maintenance titration based on the assessment of delta, alpha, and beta band activity for the pediatric population, including neonates [37].

The geriatric brain often exhibits distinct EEG age-related changes, typically associated with slightly higher qEEG values under GA [8,35,38]. Thus, corroboration of adequate DoA requires assessment of other parameters, such as a DSA signature congruent with GA status. It is crucial to distinguish the physiological baseline of relative alpha wave power attenuation from the acute “alpha wave dropout” associated with nociception, thus requiring an assessment of the patient's baseline DSA pattern during the maintenance phase, devoid of other interference sources.

Upon identification of any potential artifact, an intervention for its elimination should be performed whenever feasible and desirable. If artifact removal is impractical, then at least this should be acknowledged in any modification of the current anesthetic management plan.

## Conclusions

Several interference factors can significantly skew qEEG index values, potentially leading to misinterpretations of DoA. Acknowledging the complex nature of EEG monitoring and recognizing potential sources of artifacts and pitfalls is crucial for a more reliable depth of anesthesia monitoring. The proposed mnemonic “ANESTHESIA” offers a memorable framework to screen for potential EEG monitoring interference sources and pitfalls. This serves as a helpful tool to navigate the limitations of current monitoring technology, allowing to adjust anesthetic management accordingly, ultimately improving patient safety in the operating room.
